# Cetuximab plus chronomodulated irinotecan, 5-fluorouracil, leucovorin and oxaliplatin as neoadjuvant chemotherapy in colorectal liver metastases: POCHER trial

**DOI:** 10.1038/sj.bjc.6605940

**Published:** 2010-10-19

**Authors:** C Garufi, A Torsello, S Tumolo, G M Ettorre, M Zeuli, C Campanella, G Vennarecci, M Mottolese, I Sperduti, F Cognetti

**Affiliations:** 1 Department of Medical Oncology, Regina Elena Cancer Institute, Rome, Italy; 2 Department of Medical Oncology, S Maria degli Angeli Hospital, Pordenone, Italy; 3 Department of Surgery, San Camillo Forlanini Hospital, Rome, Italy; 4 Department of Pathology, Regina Elena Cancer Institute, Rome, Italy; 5 Department of Epidemiology and Biostatistics, Regina Elena Cancer Institute, Rome, Italy

**Keywords:** cetuximab, colorectal liver metastases, neoadjuvant chemotherapy, chronotherapy, liver resectability

## Abstract

**Background:**

We assessed the effectiveness of cetuximab plus chronomodulated irinotecan, 5-fluorouracil (5-FU), leucovorin (FA) and oxaliplatin (L-OHP) (chrono-IFLO) administered as neoadjuvant chemotherapy to increase the resectability of colorectal liver metastases.

**Methods:**

This was a phase II prospective trial with rate of liver metastases resection as primary end point. Forty-three patients with unresectable metastases were enroled: 9 with metastases >5 cm; 29 with multinodular (>4) disease; 1 with hilar location; 4 with extrahepatic lung disease. Treatment consisted of cetuximab at day 1 plus chronomodulated irinotecan 5-FU, FA and L-OHP for 2–6 days every 2 weeks. After the first 17 patients, doses were reduced for irinotecan to 110 mg m^−2^, 5-FU to 550 mg m^−2^ per day and L-OHP to 15 mg m^−2^ per day.

**Results:**

Macroscopically complete resections were performed in 26 out of 43 patients (60%) after a median of 6 (range 3–15) cycles. Partial response was noticed in 34 patients (79%). Median overall survival was 37 months (95% CI: 21–53 months), with a 2-year survival of 68% in the entire population, 80.6% in resected patients and 47.1% in unresected patients (*P*=0.01). Grade 3/4 diarrhoea occurred in 93% and 36% of patients before and after dose reduction.

**Conclusion:**

Cetuximab plus chrono-IFLO achieved 60% complete resectability of colorectal liver metastases.

Surgery can offer the potential for cure in patients with liver metastases from colorectal cancer (CRC). However, ∼80% of these patients present with initially unresectable metastatic liver disease (Adam *et al*, 2004). Historically, patients with unresectable colorectal liver metastases received chemotherapy with palliative intent but there is growing evidence that neoadjuvant chemotherapy can downsize tumours and facilitate potentially curative resection (Bismuth *et al*, 1996; Adam *et al*, 2004; Folprecht *et al*, 2005; Alberts *et al*, 2005). In a cohort of 1439 consecutive patients, 12.5% of patients with initially unresectable colorectal liver metastases were rescued to liver surgery following treatment with 5-fluorouracil (5-FU), leucovorin (FA) plus irinotecan (CPT-11) or oxaliplatin (L-OHP) or both, with a 5-year survival rate of 33% (Adam *et al*, 2004). In the past few years, we and others have developed a chronomodulated infusion schedule of CPT-11 plus 5-FU/ FA and L-OHP (chrono-IFLO) with the aim of optimise delivering of all active drugs together based on circadian tolerability (Granda *et al*, 2002; Garufi *et al*, 2003; Gholam *et al*, 2006; Garufi *et al*, 2007; Bouchahda *et al*, 2009). Indeed the tolerability and the efficacy of chemotherapeutic drugs, as well as of radiotherapy, vary 50% of more as a function of dosing time in mice or rats (Lévi, 2001).

Cetuximab is an IgG1 monoclonal antibody that specifically targets the epidermal growth factor receptor (EGFR) and that competitively blocks the binding of epidermal growth factor and other ligands, inhibiting the cellular pathways involved in the processes of cellular proliferation, angiogenesis and metastasis (Ciardiello and Tortora, 2008). Detection of positive EGFR expression by immunostaining does not reliably predict clinical outcome in patients receiving cetuximab; however, mutations in the genes of the signaling effectors downstream of EGFR, such as *KRAS*, are central to the progression of CRC and have emerged as important predictive markers of resistance to cetuximab treatment (Siena *et al*, 2009). The addition of cetuximab to doublet chemotherapy regimens such as FOLFIRI (5-FU, FA, CPT-11) or FOLFOX (5-FU, FA, L-OHP) has been shown to increase response rates, and prolong progression-free survival (PFS) and overall survival (OS) times in the first-line treatment of patients with metastatic CRC and *KRAS* wild-type tumours (Bokemeyer *et al*, 2009; Van Cutsem *et al*, 2009). In these trials, the resectability rate for liver metastases was improved by the addition of cetuximab to chemotherapy, when retrospectively evaluated.

The objective of this phase II pre-operative chemotherapy hepatic resection (POCHER) trial was to investigate the resection rate of patients with colorectal liver metastases considered unresectable following neoadjuvant treatment with cetuximab plus chrono-IFLO

## Patients and methods

### Patients

**Figure 1:**
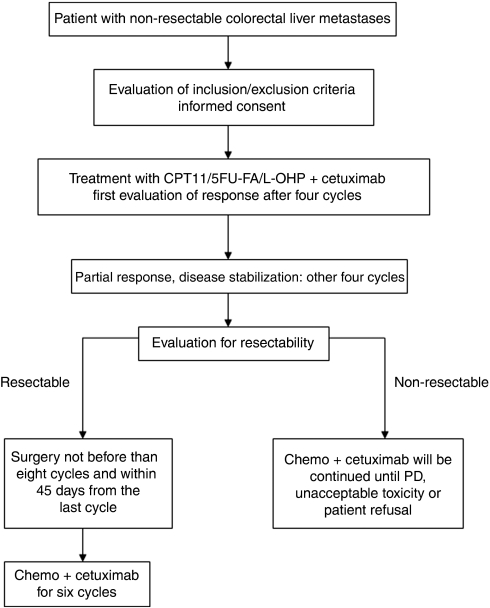
Trial design. 5-FU, 5-fluorouracil; CPT11, irinotecan; FA/L, levo-leucovorin; L-OHP, oxaliplatin; PD, progressive disease.

In all, 43 patients referred to our centres (26 patients to Regina Elena Cancer Institute and 17 patients to S Maria degli Angeli Hospital) between July 2006 and September 2008 were enrolled in this study; they presented with histologically confirmed colorectal adenocarcinoma, Eastern Cooperative Oncology Group Performance Status score of 0–1, age ⩾18 and ⩽75 years, and a life expectancy of ⩾3 months. Patients were considered unsuitable for resection of their liver metastases according to the following criteria: size larger than 5 cm, multinodular disease (>4), hilar location and presence of extrahepatic disease (except micronodular limited lung metastases). Patients with >3 metastases who had received chemotherapy to stabilise their liver disease before surgery were considered eligible for the trial as well as patients who presented with large liver metastases at the time of resection of a primary extraperitoneal rectal cancer. Assessment of liver involvement was performed by combined use of spiral computed tomography (CT) and ultrasound, which had to be performed within 1 month of the beginning of treatment. Magnetic resonance imaging, positron emission tomography/CT scans and intraoperative ultrasound were used if necessary. All staging procedures were conducted in the participating centres and all cases were discussed for eligibility after consultation with medical oncologists, radiologists and surgeons. Other inclusion criteria were adequate haematological, liver and renal function. Exclusion criteria were brain and bone metastases, whereas micronodular limited lung disease could be admitted, considering the possibility of chemotherapy control followed by resection if needed; radiotherapy within 4 weeks before the study entry; previous exposure to anti-EGFR monoclonal antibodies; clinically relevant coronary artery disease or history of a myocardial infarction in the last 12 months, acute or sub-acute intestinal occlusion or history of the inflammatory bowel disease; or any concurrent malignancy other than non-melanoma skin cancer or cervix *in situ* carcinoma. The protocol was approved by the local ethic committees of the individual centres and was registered with Eudaract number 2005-006205-28. All patients provided written informed consent. The trial design is outlined in Figure 1.

### Treatment

On day 1 of each 14-day cycle, cetuximab was infused at an initial dose of 400 mg m^−2^ and then 250 mg m^−2^ weekly. Irinotecan 130 mg m^−2^ was given on day 2 as a 6-h chronomodulated infusion, peak time at 1300 hours. From day 3–6 all patients received a 4-day chronomodulated infusion of 5-FU 600 mg m^−2^ per day and levo-leucovorin 150 mg m^−2^ per day from 2215 hours to 0945 hours, with peak delivery at 0400 hours and oxaliplatin 20 mg m^−2^ per day from 1015 hours to 2145 hours, with peak delivery at 1600 hours. Treatment was administered using a four-reservoir, multichannel, programmable in-time pump (Melodie, Aguettant, France) in an outpatients setting. An interim analysis for toxicity was performed after the first 17 patients had been treated and dose reductions were implemented, such that all subsequent patients received irinotecan 110 mg m^−2^, 5-FU 550 mg m^−2^ per day and oxaliplatin 15 mg m^−2^ per day. In the event of predefined toxic effects related to chemotherapy or cetuximab, protocol-specified treatment modifications were allowed.

Tumour response was assessed every four cycles according to Response Evaluation Criteria In Solid Tumours (RECIST) (Therasse *et al*, 2000). Resection was evaluated and intended to be performed after eight cycles and within 45 days of the last treatment cycle. Patients with unresectable tumours continued treatment until disease progression, unacceptable toxicity or patient refusal. Following resection, patients were continued on treatment for an additional six cycles.

### Assessments

The primary end point was resection rate of liver metastases, which was evaluated in all patients. Secondary end points were rate of complete pathologic responses, response rates, PFS and OS. Response rate was calculated based on RECIST for the intention-to-treat population, which included all patients who had received at least one course of therapy. The PFS was calculated for all patients from the day of study entry until the date of progression of disease. Time to relapse was the interval between surgery and the first recurrence of disease. Patients who did not progress were censored at the last date they were known to be alive. Patients who died of disease and for whom a date of progression was not available were considered to have progressed on the day of their death. Safety was an additional secondary end point, and all toxicities episodes were graded according to the National Cancer Institute Common Toxicity Criteria Version 3.

### Surgery

Limited or major hepatic resection was considered for the analysis only when the extent of resection was complete (R0). The R1 (margin positive) and R2 (unresectable) resections were recorded. Ablative techniques were allowed in addition to surgery in the operative room.

### The EGFR and KRAS testing

EGFR expression was assessed by immunohistochemistry, but was not an eligibility criterion. It was evaluated by EGFR-pharmDx-kit (Dako, Milan, Italy) and was scored considering percentage of staining with a cut off of 10% of cells.

The effect of *KRAS* mutations on cetuximab activity was not known at the time the study was designed; tumour *KRAS* mutation status was subsequently assessed retrospectively on patient tumour samples by direct sequencing.

### Statistical analysis

The sample size calculation was based on the two-step Simon minimax design. Chrono-IFLO plus cetuximab would be considered ineffective and the trial would be stopped if the resection rate was ⩽10%. Chrono-IFLO plus cetuximab would be considered effective and the study would be pursued if the resection rate was ⩾25%. On the basis of an α level of 5% and a power of 80%, a minimum of 22 subjects had to be enrolled during the first step of the study and 18 subjects during the second step (40 subjects overall). The PFS and OS were calculated based on Kaplan–Meier curves. Differences in toxicity before and after dose reduction were calculated by the McNemat test for paired data.

## Results

A total of 43 patients were enrolled and evaluated (Table 1). Median age was 61 (range 33–75) years and the majority of patients were male (63%). Most patients had undergone resection of their primary tumour (90%) and most had synchronous liver disease (81%). Multinodular involvement of >4 lesions was the predominant reason for unresectability (68%). Four patients had extrahepatic limited lung disease. Of the 37 patients evaluable for tumour *KRAS* mutation status, 81% had *KRAS* wild-type tumours. In six patients it was not possible to collect tumour samples.

After an interim analysis in the first 17 patients, doses were reduced because of the unacceptable toxicity. The reduction for all patients occurred within the third cycle. One case of sepsis was observed, one patient had severe cardiac toxicity with arrhythmias leading to treatment interruption, and another patient developed renal failure. One of the 17 patients refused to continue therapy because of the toxicity. Diarrhoea was the major treatment toxicity reaching grade 3/4 in 93% patients, and was often accompanied with abdominal pain (33% of patients; Table 2). Grade 2/3 afebrile neutropenia was found in 19% of patients. After dose reduction, there was a significant reduction in the proportion of patients who experienced diarrhoea, although grade 3/4 diarrhoea was still present in more than one-third of patients and one-quarter had grade 2 diarrhoea (Table 2). There were numerically fewer cases of grade 2–3 neutropenia after dose reduction (19 *vs* 13%) and no relevant thrombocytopenia or anaemia was observed. Grade 2–3 sensory neuropathy was not recorded in any of the 43 patients.

**Figure 2:**
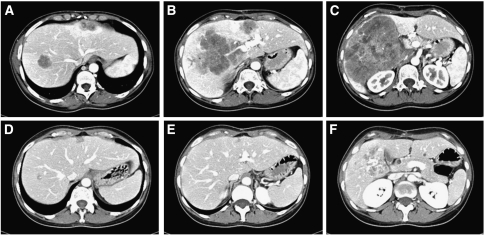
Pre-treatment (**A**, **B**, **C**) and after six courses (**D**, **E**, **F**) spiral TC-scan of a patient submitted to a two-step hepatectomy (she is free of disease after 34 months of follow-up).

All 43 enroled patients were evaluated for response and resectability. Partial responses were obtained in 34 patients, with an objective response rate of 79.1% (95% CI: 66.9–91.2%). All resected patients had obtained a partial response. In all, 26 patients underwent radical liver surgery after neoadjuvant chemotherapy with cetuximab plus chrono-IFLO, with a R0 resection rate of 60% (95% CI: 45.8–75.1) fulfilling the primary end point. Two patients underwent R1 resection and six patients (14%) underwent explorative laparoscopy and disease was judged unresectable (R2). Of the 26 patients who underwent radical surgery, 13 (30% of all patients) had multiple wedge resections, 9 (21%) underwent two-step hepatectomies, 3 (7%) had right hepatectomies and 1 (2%) had a left hepatectomy. Two patients received radiofrequency ablation in addition to surgery. Two complete pathological responses were observed in the resected specimens. Stable disease was achieved in five patients, three patients discontinued because of the toxicity and one refused to continue before study evaluation. The median number of cycles per patient was 10 (range 2–18). The median before surgery was 6 (range 3–15) rather than the 8 courses planned, because of the rapid tumour shrinkage (Figure 2). The median time from last course of chemotherapy to surgery was 5 weeks (range 1–11). The median number of cycles after surgery was 5 (range 1–6). Median time of treatment for all patients was 28 weeks (range 2–59). A total of 20 patients completed the 14 cycles of treatment planned in the trial design. Dose reduction did not affect response rate (76.5% in the first 17 patients *vs* 80.8% in subsequent patients enroled, *P*=0.73) and resection rate (62.6 *vs* 58.8%, *P*=0.47). Moreover toxicity did not affect time to surgery (*P*=0.23).

Regarding the four patients with extrahepatic lung disease, only one was resected for liver and lung metastases, one rapidly progressed in the lung after liver resection and two were never resected.

At a median follow-up of 22 months (range 1–43) there were 7 out of 43 (16%) patients alive without recurrence, 17 out of 43 (39.5%) alive with recurrence, two patients lost to follow-up and 17 out of 43 (39.5%) patients who died because of the disease progression. Among seven patients alive without recurrence, five patients were KRAS wild type representing the 16.6% (5 out of 30) of all KRAS wild-type patients, whereas two patients were KRAS muted.

**Figure 3:**
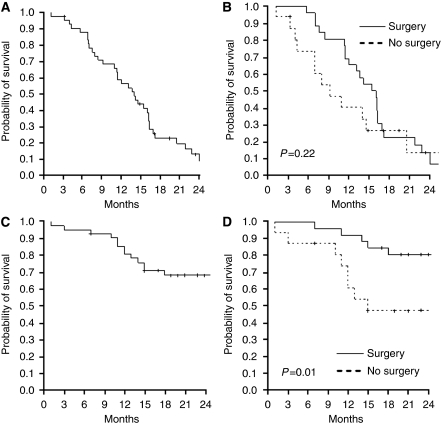
Kaplan–Meier curves of progression-free survival (PFS) and overall survival. (**A**) The PFS in the entire population (*n*=43); (**B**) PFS in resected (unbroken line) and not resected (broken line) patients. (**C**) Overall survival in the entire population (*n*=43); (**D**) overall survival in resected (unbroken line) and not resected (broken line) patients.

The PFS for all patients was 14 months (95% CI: 11–17 months; Figure 3). For those patients who were resected, PFS was 15 months (95% CI: 12–19 months; Figure 3), whereas PFS was 9 months (95% CI: 1–17 months) for those patients who were not resected. The median time from surgery until relapse was 11 months (95% CI: 9–13 months). After liver surgery, 10 out of 26 patients (38%) had disease recurrence in the liver, 4 out of 26 (15%) patients had disease recurrence outside the liver (one presented already extrahepatic disease at study entry) and 6 out of 26 patients (23%) had disease recurrence both in the liver and outside (none of these 6 patients presented extrahepatic disease at study entry). Median estimated OS for all patients was 37 months (95% CI: 21–53 months), with 68.2% of patients alive at 2 years in the entire population, 80.6% in resected patients and 47.1% in non-resected patients (*P*=0.01; Figure 3).

In those patients with liver-only recurrent disease, it was possible to perform a re-hepatectomy in 5 patients; 19 patients received second-line chemotherapy and 7 patients received third-line chemotherapy. Bevacizumab plus FOLFIRI or FOLFOX was used in 12 patients as second- or third-line therapy. Seven patients received FOLFOX or XELOX (capecitabine plus oxaliplatin); various other treatments including locoregional therapies were used in the remaining patients.

## Discussion

This is the first study that tests the relevance of cetuximab in addition to a triplet combination as first-line treatment for metastatic colorectal cancer and uses the rate of macroscopic resection of liver metastases as main end point.

In the POCHER trial, we showed that intensive chemotherapy with chrono-IFLO plus cetuximab resulted in high response rates, rapid tumour shrinkage and a resection rate of 60%. In addition, the combination produced an estimated median OS for all patients of 37 months after a median follow-up of 22 months. The main treatment toxicity was severe diarrhoea, which affected most of the patients before dose reduction and was still present in more than one-third of patients after dose reductions. Neutropenia was not common and neurotoxicity was absent. Dose reduction due to toxicity did not compromise response rate, resection rate and time to surgery.

The results of the POCHER trial extend the findings of the recent CELIM trial of cetuximab in combination with either FOLFOX (*n*=56) or FOLFIRI (*n*=55) as neoadjuvant therapy in patients with unresectable colorectal liver metastases (Folprecht *et al*, 2010). Resection rates were 38% with cetuximab plus FOLFOX and 30% with cetuximab plus FOLFIRI, with a confirmed partial or complete response noticed in 68 and 57% of the patients, respectively. Toxicity was acceptable in the CELIM trial, with 72% of all patients experiencing at least one episode of grade 3/4 toxicity; 23% of patients experienced grade 3/4 neutropenia and 14% experienced grade 3/4 diarrhoea. Other studies have provided evidence of the effectiveness of cetuximab plus doublet chemotherapy for unselected populations of patients with advanced CRC. In the CRYSTAL trial, the addition of cetuximab to FOLFIRI showed an increase in the resection rate from 4.5 to 9.8% in a subgroup of patients with liver-limited disease (Van Cutsem *et al*, 2009). In the OPUS study, in which FOLFOX±cetuximab was used, the resection rate for liver metastases doubled from 2.4 to 4.7% in the cetuximab group (Bokemeyer *et al*, 2009).

The use of a triplet rather than a doublet chemotherapy schedule was justified by the results obtained by Falcone *et al* (2007) in a randomised study comparing triplet FOLFOXIRI chemotherapy with FOLFIRI, in the absence of cetuximab. They found the resection rate to be greater in the FOLFOXIRI arm (36% *vs* 12% *P*=0.017) in patients with metastases confined to the liver. Treatment with FOLFOXIRI resulted in a 5-year survival rate of 42%. Recently the same group added bevacizumab to the FOLFOXIRI regimen with 40% resection rate of liver metastases in an unselected population. Table 3 compares our data with other relevant experiences in this field.

One possible explanation for the high activity of cetuximab plus chemotherapy in the treatment of patients with liver metastases could be the high incidence of patients with *KRAS* wild-type tumours; 70 and 80% in the CELIM and POCHER trials, respectively, which is higher than the ∼60% usually observed in CRC patient populations. This point warrants further validation in a patient population with liver only metastases. Other potential reasons, such as a greater immunological effect of cetuximab in the liver, could be speculated.

Another feature of this trial was the use of a chronomodulated schedule. The EORTC trial 05963 comparing chronomodulated *vs* conventional delivery of FOLFOX showed male patients receiving a chronomodulated regimen to have a higher response rate and longer PFS and OS times *vs* conventional delivery; however, women had significantly longer PFS and OS using conventional delivery (Giacchetti *et al*, 2006). Interestingly, in recent meta-analysis of three randomised trials the rate of complete macroscopic resections of liver metastases was 12.5% in men using chronomodulated delivery *vs* 7.8–8.5% in men using conventional delivery, or in women on either schedule (Levi *et al*, 2009). In an unselected series of patients treated with chrono-IFLO plus cetuximab, we observed more than 20% dose reduction due to diarrhoea in 47% of females *vs* 12% of males (*P*=0.03) (Garufi *et al*, 2010). To increase tolerability of this schedule, we could consider irinotecan pharmacogenetics, identification of better circadian peaks for women and delivering of treatment every 3 weeks in frail patients. Moreover, we could not preclude the use of intra-arterial ‘adjuvant’ chemotherapy as postoperative treatment to reduce intrahepatic recurrence rate.

A randomised phase II trial with cetuximab plus FOLFOX or FOLFIRI *vs* chrono-IFLO and a different trial testing various chronotherapeutic schedules could helpful to better define our strategy and chronobiological profile.

In conclusion, in the POCHER trial, the combination of an intensive chemotherapy regimen of chronomodulated IFLO plus cetuximab was associated with high response rates and facilitated radical resections in 60% of patients with colorectal liver metastases, providing improved long-term survival.

## Figures and Tables

**Table 1:** Patient characteristics at baseline

	**No. of patients**	**%**
Entire population	43	100
Median (range) age (years)	61 (33–75)	
Male/female	27/16	63/37
Colon/rectum	34/9	79/21
Primary tumour resected	39	90
Synchronous metastases	35	81
Liver involvement >25%	34	79
		
*Unresectability*		
Size >5 cm	9	21
Multinodular >4	29	68
Hilar location	1	2
Extrahepatic disease	4	9
Median (range) CEA, ng ml^−1^	55 (1–6600)	
Median (range) CA19-9, U l^−1^	92 (2–66440)	
		
*EGFR (extent of staining)*		
0	8	23
1	4	11
2	18	52
3	5	14
*KRAS* wild type/mutant^a^	30/7	81/19

Abbreviations: CA19–9=carbohydrate antigen 19–9; CEA=carcinoembryonic antigen; EGFR=epidermal growth factor receptor.

a *n*=37

**Table 2:** Major grade 2–4 toxicities before and after dose reductions

	**Patients experiencing toxicity (%)**			
**Type of toxicity**	**Grade**	**Before dose reduction**	**After dose reduction**	***P*-value**
Diarrhoea	2	6	26	NS
	3	81	35	0.005
	4	13	1	0.006
Abdominal pain	2	31	25	NS
	3	2	7	0.05
	4	0	0	NS
Fatigue	2	43	37	NS
	3	8	12	NS
	4	2	0	NS
Nausea/vomiting	2	50	44	NS
	3	12	10	NS
	4	1	0	NS
Afebrile neutropenia	2	12	7	NS
	3	7	6	NS
Cutaneous rash	2	50	66	NS
	3	20	15	NS

Abbreviation: NS=not significant.

**Table 3:** Chemotherapy trials of neoadjuvant chemotherapy for colorectal cancer liver metastases

	**Schedule**	**Selected patients**	**No. of patients**	**RR (%)**	**R0 resection (%)**
Cetuximab plus doublets	Cetuximab+FOLFIRI (Van Cutsem, *et al*, 2009)	No	132	46.9	4.8
	Cetuximab+FOLFOX4 (Bokemeyer *et al*, 2009)	No	169	46.0	4.7
	Cetuximab+FOLFOX or FOLFIRI (Folprecht *et al*, 2010)	Yes	111	85.0 (Oxa)-66.0 (CPT)	34
Triplets	FOLFOXIRI (Falcone *et al*, 2007)	No	39	66	36
	FOLFOXIRI (De la Cámara *et al*, 2004)	Yes	39	64	43
	FOLFOXIRI (Ychou *et al*, 2008)	Yes	34	70	26
Monoclonal antibody plus triplets	Cetximab+chrono-IFLO (Garufi *et al*, present study)	Yes	43	79.1	60
	Bevacizumab+FOLFOXIRI (Masi *et al*, 2010)	No	30	80	40

Abbreviations: CPT=irinotecan; Oxa=oxaliplatin; RR=response rate; R0 resection=radical resection.
